# Mycobacterial Cell Wall Synthesis Inhibitors Cause Lethal ATP Burst

**DOI:** 10.3389/fmicb.2018.01898

**Published:** 2018-08-15

**Authors:** Annanya Shetty, Thomas Dick

**Affiliations:** ^1^Department of Medicine, Yong Loo Lin School of Medicine, National University of Singapore, Singapore, Singapore; ^2^Department of Microbiology and Immunology, Yong Loo Lin School of Medicine, National University of Singapore, Singapore, Singapore; ^3^Public Health Research Institute, New Jersey Medical School, Rutgers, The State University of New Jersey, Newark, NJ, United States

**Keywords:** *Mycobacterium*, isoniazid, ethambutol, bedaquiline, *iniBAC*

## Abstract

Mycobacterial cell wall inhibitors interfere with targets involved in synthesis of mycolic acids, arabinogalactan and peptidoglycan. These antibiotics corrupt structural integrity of the cell envelope and this is believed to be the cause of drug mediated cell death. Here, we show that treatment of *Mycobacterium bovis* BCG with these mechanistically different classes of cell wall inhibitors at MIC caused a 4 to 5-fold increase in intrabacterial ATP concentration. This effect on ATP homeostasis was specific to inhibitors of cell wall synthesis and not observed for other anti-tuberculosis drugs. Treating *M. bovis* BCG with sub-MIC concentrations of the ATP synthase inhibitor bedaquiline or the uncoupler carbonyl cyanide 3-chlorophenylhydrazone suppressed drug induced ATP surge, suggesting that the increase in ATP concentration was due to increased oxidative phosphorylation. Pharmacological suppression of the ATP burst attenuated bactericidal activity of the cell wall-targeting drugs up to 100-fold, suggesting that increased ATP levels are associated with the lethal effect of these antibiotics. Interestingly, inhibition of the ATP burst also suppressed induction of the promoter of the cell envelope stress response operon *iniBAC* by cell wall inhibitors suggesting a link between ATP surge and *iniBAC* expression. In conclusion, we show that treatment of *M. bovis* BCG with inhibitors of cell wall synthesis causes a burst of intrabacterial ATP by increasing oxidative phosphorylation. This ATP surge appears to be required for induction of the *iniBAC* cell envelope stress response operon and to contribute to drug induced cell death. Hence, this work revealed links between inhibition of cell wall synthesis, oxidative phosphorylation, *iniBAC* induction and cell death. The identification of the molecular mechanisms linking these processes may reveal novel targets for the discovery of bactericidal antibiotics.

## Introduction

Mycobacteria cause a range of infectious diseases. Most prevalent is tuberculosis lung disease caused by *Mycobacterium tuberculosis* resulting in more than a million deaths per year (García-Basteiro et al., 2018). In addition to tuberculosis, lung disease caused by so-called non-tuberculous mycobacteria (NTM), including *Mycobacterium abscesuss*, is increasing (Wu et al., 2018). Mycobacterial infections are difficult to eradicate due to intrinsic and acquired drug resistance (Wu et al., 2018). New drugs are urgently needed to improve the treatments of mycobacterial diseases. For the discovery of new drugs novel targets are needed. One approach to identify new targets is to determine the exact cellular mechanisms how our current antibiotics cause bacterial death.

Anti-mycobacterials modulate well characterized primary targets involved in a range of pathways including cell wall synthesis, oxidative phosphorylation, co-factor biosynthesis, and nucleic acid and protein synthesis (Koul et al., 2011). However, how modulation of primary targets results in the death of mycobacteria is largely unknown (Dick and Young, 2011). Studies of other bacteria (Belenky et al., 2015; Lobritz et al., 2015; Yang J.H. et al., 2017) and more limited studies in mycobacteria (Baek et al., 2011; Koul et al., 2014; Nandakumar et al., 2014; Maglica et al., 2015; Vilcheze et al., 2017) have shown that primary target modulation by antibiotics is often followed by profound changes in metabolism which contribute to the bactericidal activity of the drugs.

ATP homeostasis is critical for maintaining viability of bacteria. The new anti-tuberculosis drug BDQ, an inhibitor of F-ATP synthase, shows that reducing the bacterial ATP level below a threshold concentration causes cell death (Andries et al., 2005; Rao et al., 2008; Gengenbacher et al., 2010). We speculated that there may also be an upper threshold concentration of ATP above which the molecule exerts toxic effects. In support of our hypothesis, Tomioka and colleagues demonstrated recently that exogenously supplied ATP has antimycobacterial activity (Tatano et al., 2015). Here, we asked whether treatment with anti-tuberculosis drugs is associated with a toxic increase of intrabacterial ATP levels.

## Materials and Methods

### Strain, Media, and Drugs

*Mycobacterium bovis* BCG Pasteur (ATCC #35734) was obtained from the American Type Culture Collection. *M. bovis* BCG Pasteur (ATCC #35734) p*iniBAC*-RFP harbors the coding sequence of the red fluorescent protein mCherry under control of the *iniBAC* promoter and was constructed as previously described (Yang T. et al., 2017). Liquid cultures were grown in complete Middlebrook 7H9 medium (BD Difco) supplemented with 0.05% Tween 80, 0.4% glycerol, and 10% albumin-dextrose-catalase enrichment (Becton Dickinson) at 37°C and 80 rpm. To grow cultures on solid medium for CFU determination Middlebrook 7H11 agar (BD Difco) supplemented with 0.2% glycerol and 10% oleic-acid-albumin-dextrose-catalase enrichment was used. Drugs and chemicals were from Sigma-Aldrich, with the exception of BDQ which was from MedChem Express. All drugs were dissolved in 90% dimethyl sulfoxide and filter sterilized to prepare stocks that were kept at -20°C.

### Minimum Inhibitory Concentration Determination

Minimum inhibitory concentrations (MIC) were determined by the broth microdilution method (Wiegand et al., 2008). Exponentially growing precultures were seeded in clear 96-well flat-bottom plates (Greiner Bio-One) at OD_600_ = 0.05 in the presence of two-fold serial dilutions of assay compounds in a volume of 200 μl/well. Assay plates were sealed (Breath-Easy membrane, Sigma-Aldrich) and incubated for 7 days at 37°C and 80 rpm prior to turbidity determination (OD_600_, Tecan M200Pro plate reader). Percentage growth was determined compared to untreated control and plotted as a function of drug concentration (Graph Pad Prism 5 software). The concentrations that inhibit 90% of growth were recorded as MIC.

### Determination of Bactericidal Effects

Cidal effects of drugs were determined by CFU enumeration on agar plates after exposure to stated concentrations of test compounds as described previously (Yang T. et al., 2017). Briefly, appropriately diluted mid log phase precultures of *M. bovis* BCG were treated for 24 h with stated concentrations of drugs. Treated cultures were then diluted and plated on agar. Colonies were counted after 2–3 weeks of incubation at 37°C.

### Intrabacterial ATP Measurement

ATP was quantified using the BacTiter-Glo^TM^ Microbial Cell Viability Assay kit from Promega according to the manufacturer’s instructions and as previously described (Gengenbacher et al., 2010). Briefly, *M. bovis* BCG cultures were grown in Middlebrook 7H9 broth to mid log phase, diluted and treated with various drugs for 24 h. The assay time was kept short (< / = one doubling time) to minimize effects of growth (or drug induced cell death). OD_600_ of cultures was measured at the beginning and end of the experiments and the observed OD_600_ increase was < / = 2-fold as expected. 25 μl of samples (total culture) were mixed with an equal volume of freshly prepared BacTiter-Glo^TM^ reagent in white flat bottomed 96 well plates (Corning) and lysis was carried out for 10 min at room temperature with shaking at an amplitude of 3 mm inside the M200Pro plate reader (Tecan). Emitted luminescence was displayed as relative light units (RLU) as described previously (Gengenbacher et al., 2010).

### *iniBAC* Promoter Activity Measurement

For measurement of activity of the *iniBAC* promoter, the reporter strains *M. bovis* BCG p*iniBAC*-RFP, described previously (Yang T. et al., 2017) was used. Briefly, appropriately diluted mid log phase precultures of *M. bovis* BCG p*iniBAC*-RFP were exposed to stated concentrations of drugs for 24 h. Fluorescence was then measured on a M200Pro plate reader (λ587/630 nm) and displayed as relative fluorescence unit (RFU) as described previously (Yang T. et al., 2017).

## Results

### Cell Wall Synthesis Inhibitors Cause a Burst of Intrabacterial ATP Concentration

**FIGURE 1:**
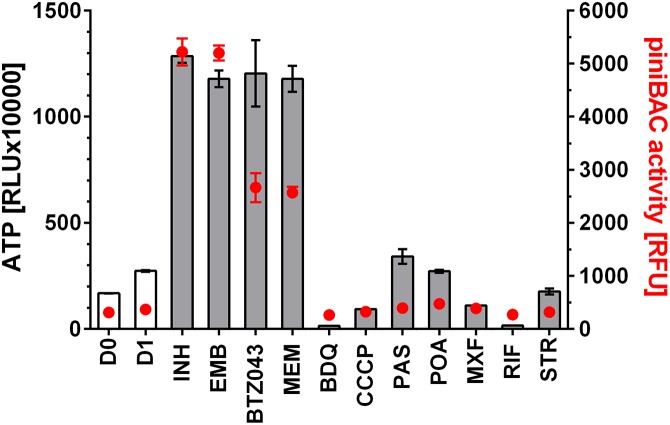
Effect of treatment of *M. bovis* BCG p*iniBAC*-RFP with anti-mycobacterials on intrabacterial ATP level and *iniBAC* promoter activity. Exponentially growing *M. bovis* BCG p*iniBAC*-RFP cultures were treated with cell wall inhibitors that interfere with mycolic acid synthesis (INH), arabinogalactan synthesis (EMB, BTZ043) or peptidoglycan synthesis (MEM), and with inhibitors of oxidative phosphorylation (BDQ, CCCP), cofactor synthesis (PAS, POA) and nucleic acid and protein synthesis (MXF, RIF, STR) at the MIC of the respective drugs for 24 h. Then ATP content was measured with the BacTiter-Glo^TM^ assay (“ATP,” gray bars, in relative light units, RLU). *iniBAC* promoter activity was measured by determining the fluorescence of the cultures (“p*iniBAC* activity,” red circles, in relative fluorescence units, RFU). D0 and D1 show the RLU and RFU values at the start and the end of the experiment for drug free cultures. For abbreviations of drugs and their targets see **Table 1**. Experiments were carried out three times independently in duplicates. Mean values and standard deviations for one representative experiment are shown. The ATP content determinations were also carried out for *M. bovis* BCG not carrying the p*iniBAC*-RFP reporter and found to be the same as the values obtained for *M. bovis* BCG p*iniBAC*-RFP.

To determine whether any of the anti-mycobacterial drugs cause an increase in intrabacterial ATP concentration, we treated exponentially growing *M. bovis* BCG with 11 different drugs including inhibitors of cell wall-, cofactor-, nucleic acid- and protein-synthesis at their MIC concentrations (**Table 1**). After 24 h, ATP content of the cultures was determined with the BacTiter-Glo^TM^ assay with RLU as readout. Surprisingly, we not only found that some drug treatments resulted in a 4 to 5-fold increase of intrabacterial ATP levels, but that all cell wall synthesis inhibitors, independent of the nature of their primary target, had this effect (**Figure 1**). In contrast, all tested non-cell wall synthesis inhibitors had either no effect on ATP levels or, in the case of inhibitors of oxidative phosphorylation, reduced intrabacterial ATP concentration as expected (**Figure 1**). These results show that treatment of *M. bovis* BCG with different classes of cell wall synthesis inhibitors cause an increase in intrabacterial ATP concentration, not observed upon exposure to non-cell wall inhibitors.

**Table 1:** MIC for *M. bovis* BCG and mechanism of action of anti-mycobacterials used in the study.

Drug name	MIC (μM)	Primary target	Target pathway	Pathway category	Reference
Isoniazid, INH	1.5	Enoyl acyl carrier protein reductase InhA	Mycolic acid synthesis	Cell wall synthesis	Zhang, 2005
Ethambutol, EMB	3.1	Arabinosyltransferase EmbC	Arabinogalactan synthesis		
BTZ043	0.003	Decaprenylphosphoryl-β-D-ribose oxidase DprE1			Makarov et al., 2009
Meropenem, MEM	12.5	Transpeptidase Ldt_Mt2_	Peptidoglycan synthesis		Kim et al., 2013
Bedaquiline, BDQ	0.26	F-ATP synthase	ATP synthesis	Oxidative phosphorylation	Andries et al., 2005
Carbonyl cyanide m-chlorophenyl hydrazine, CCCP	50	N.A.^∗^	Transmembrane proton gradient		Feng et al., 2015
Para-amino salicylic acid, PAS	3.1	Dihydrofolate reductase	Tetrahydrofolate synthesis	Cofactor synthesis	Zheng et al., 2013
Pyrazinoic acid, POA^∗∗^	1000	Aspartate decarboxylase PanD	Coenzyme A synthesis		Gopal et al., 2017
Moxifloxacin, MXF	0.4	DNA Gyrase	DNA topology	Nucleic acid synthesis	Zhang, 2005
Rifampicin, RIF	0.025	RNA Polymerase	RNA synthesis		
Streptomycin, STR	0.2	16S rRNA	Protein synthesis	Protein synthesis	


*^∗^Not applicable; CCCP is a protonophore and therefore has no primary macromolecular target. ^∗∗^POA is the bioactive metabolite of prodrug pyrazinamide.*

### Inhibitors of Oxidative Phosphorylation Prevent the Drug-Induced ATP Burst

**FIGURE 2:**
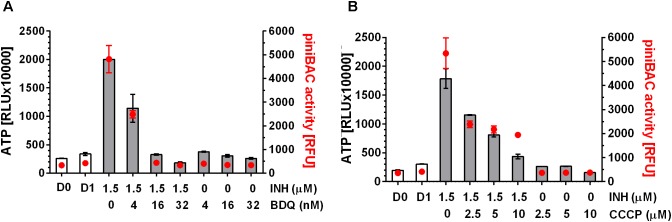
Effect of co-treatment of *M. bovis* BCG p*iniBAC*-RFP with cell wall synthesis inhibitor INH and inhibitors of oxidative phosphorylation on ATP level and p*iniBAC* activity. **(A)** Exponentially growing *M. bovis* BCG p*iniBAC*-RFP cultures were co-treated with the mycolic acid synthesis inhibitor INH at MIC (1.5 μM) and increasing sub-MIC concentrations of the F-ATP synthase inhibitor BDQ for 24 h (MIC BDQ = 0.26 μM). Then ATP content was measured with the BacTiter-Glo^TM^ assay (“ATP,” gray bars, in relative light units, RLU). *iniBAC* promoter activity was measured by determining the fluorescence of the cultures (“p*iniBAC* activity,” red circles, in relative fluorescence units, RFU). D0 and D1 show the RLU and RFU values at the start and the end of the experiment for drug free cultures. **(B)** Same experiment as in **(A)** replacing the F-ATP-synthase inhibitor BDQ with the protonophore CCCP. MIC CCCP = 50 μM. Experiments were carried out three times independently in duplicates. Mean values and standard deviations for one representative experiment are shown. Note: the RLU and RFU numbers are not normalized for growth. As the assay time (24 h) was kept short (within one generation time) the maximum increase in OD_600_ (drug free) was two-fold [reflected by slight increases in RLU and RFU of drug-free samples at day 1 (D1) compared to the start of the experiment (D0)]. For all drug containing samples OD_600_ increased 1.5 to 2 fold.

Increase in intrabacterial ATP concentration could be due to reduced ATP utilization or due to increased ATP synthesis. If the ATP surge is due to increased ATP synthesis via oxidative phosphorylation, inhibiting F-ATP synthase should prevent the surge. To determine whether inhibition of ATP synthase prevents cell wall synthesis inhibitor-induced ATP increase we co-treated *M. bovis* BCG cultures with the MIC of the mycolic acid synthesis inhibitor INH, causing a surge of ATP, and increasing – but sub-MIC – concentrations of the ATP synthase inhibitor BDQ for 24 h. Co-treatment of cultures with INH at MIC with sub-MICs of BDQ indeed reduced the ATP surge in a dose dependent manner (**Figure 2A**). At 0.016 μM BDQ, a concentration which corresponds to 1/16th its MIC (0.26 μM), the ATP surge was completely suppressed (**Figure 2A**).

To provide BDQ and ATP synthase independent evidence that inhibition of oxidative phosphorylation suppresses the INH-induced ATP burst, we carried out the same experiments with a compound targeting a different component of the oxidative phosphorylation process. Co-treatment of cultures with INH and sub-MICs of the uncoupler carbonyl cyanide 3-chlorophenylhydrazone (CCCP), a protonophore which reduces the proton concentration gradient across the bacterial membrane and thus affects ATP synthesis, resulted in suppression of the ATP surge (**Figure 2B**).

**FIGURE 3:**
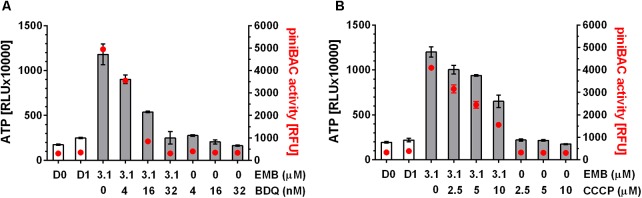
Effect of co-treatment of *M. bovis BCG* p*iniBAC*-RFP with cell wall synthesis inhibitor, EMB and inhibitors of oxidative phosphorylation on ATP level and p*iniBAC* activity. **(A)** Exponentially growing *M. bovis* BCG p*iniBAC*-RFP cultures were co-treated with the arabinogalactan synthesis inhibitor, EMB at MIC (3.1 μM) and increasing sub-MIC concentrations of the F-ATP synthase inhibitor BDQ for 24 h (MIC BDQ = 0.26 μM). Then ATP content was measured with the BacTiter-Glo^TM^ assay (“ATP,” gray bars, in relative light units, RLU). *iniBAC* promoter activity was measured by determining the fluorescence of the cultures (“p*iniBAC* activity,” red circles, in relative fluorescence units, RFU). D0 and D1 show the RLU and RFU values at the start and the end of the experiment for drug free cultures. **(B)** Same experiment as in **(A)** replacing the F-ATP-synthase inhibitor BDQ with the protonophore CCCP. MIC CCCP = 50 μM. Experiments were carried out three times independently in duplicates. Mean values and standard deviations for one representative experiment are shown. Note: the RLU and RFU numbers are not normalized for growth. As the assay time (24 h) was kept short (within one generation time) the maximum increase in OD_600_ (drug free) was two-fold (reflected by slight increases in RLU and RFU of drug-free samples at day 1 (D1) compared to the start of the experiment (D0)). For all drug containing samples OD_600_ increased 1.5 to 2 fold.

To determine whether these findings can be extended to mechanistically different cell wall synthesis inhibitors, we tested the effects of oxidative phosphorylation inhibitors on the ATP surge caused by the arabinogalactan synthesis inhibitor EMB and found the same effects: BDQ or CCCP at sub-MIC prevented EMB induced ATP increase (**Figure 3**).

These results show that sub-MIC concentrations of oxidative phosphorylation inhibitors suppress the ATP burst triggered by cell wall synthesis inhibitors. This suggest that the observed ATP surge is due to increased ATP synthesis by oxidative phosphorylation. Hence, cell wall synthesis inhibitors appear to trigger a common stress response resulting in increased oxidative phosphorylation which in turn results in increased intrabacterial ATP levels.

### Pharmacological Suppression of Drug Induced ATP Burst Attenuates Bactericidal Activity

**FIGURE 4:**
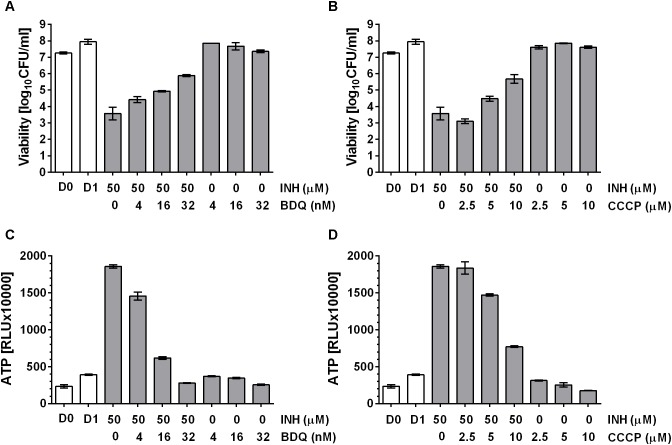
Effect of co-treatment of *M. bovis* BCG with cell wall synthesis inhibitor, INH and inhibitors of oxidative phosphorylation on viability. **(A)** Exponentially growing *M. bovis* BCG cultures were co-treated with the mycolic acid synthesis inhibitor INH at 50 μM and increasing sub-MIC concentrations of the F-ATP synthase inhibitor BDQ for 24 h (MIC BDQ = 0.26 μM). Then the viability of the culture was determined by plating and CFU count (“viability,” in CFU/ml). **(B)** Exponentially growing *M. bovis* BCG cultures were co-treated with the mycolic acid synthesis inhibitor INH at 50 μM and increasing sub-MIC concentrations of the protonophore CCCP for 24 h (MIC CCCP = 50 μM). Then the viability of the culture was determined by plating and CFU count (“viability,” in CFU/ml). **(C)** ATP content of cultures in **(A)**. **(D)** ATP content of cultures in **(B)**. ATP was measured with the BacTiter-Glo^TM^ assay (“ATP,” in relative light units, RLU). D0 and D1 show the CFU/ml and RLU values at the start and the end of the experiments for drug free cultures. Experiments were carried out three times independently in duplicates. Mean values and standard deviations for one representative experiment are shown.

**FIGURE 5:**
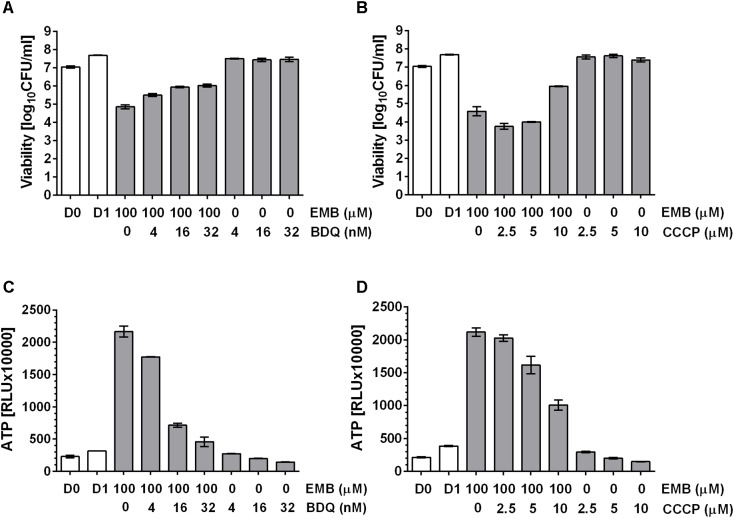
Effect of co-treatment of *M. bovis* BCG with cell wall synthesis inhibitor EMB and inhibitors of oxidative phosphorylation on viability. **(A)** Exponentially growing *M. bovis* BCG cultures were co-treated with the arabinogalactan synthesis inhibitor, EMB at 100 μM and increasing sub-MIC concentrations of the F-ATP synthase inhibitor BDQ for 24 h (MIC BDQ = 0.26 μM). Then the viability of the culture was determined by plating and CFU count (“viability,” in CFU/ml). **(B)** Exponentially growing *M. bovis* BCG cultures were co-treated with the arabinogalactan inhibitor, EMB at 100 μM and increasing sub-MIC concentrations of the protonophore CCCP for 24 h (MIC CCCP = 50 μM). Then the viability of the culture was determined by plating and CFU count (“viability,” in CFU/ml). **(C)** ATP content of cultures in **(A)**. **(D)** ATP content of cultures in **(B)**. ATP was measured with the BacTiter-Glo^TM^ assay (“ATP,” in relative light units, RLU). D0 and D1 show the CFU/ml and RLU values at the start and the end of the experiments for drug free cultures. Experiments were carried out three times independently in duplicates. Mean values and standard deviations for one representative experiment are shown.

Tomioka and colleagues (Tatano et al., 2015) showed that exogenously supplied ATP displayed antimicrobial activity against mycobacteria, suggesting an upper threshold of intrabacterial ATP concentration above which ATP becomes toxic to the cell. Thus, we hypothesized that the observed ATP surge may be toxic and contribute to the bactericidal activity of the drugs. If true, suppression of the ATP surge should attenuate the killing activity of cell wall synthesis inhibitors. *M. bovis* BCG cultures were treated with high dose INH alone or co-treated with increasing sub-MIC concentrations of the oxidative phosphorylation inhibitors BDQ or CCCP for 24 h after which CFU were enumerated by plating on agar. Whereas INH treatment alone resulted in a four log kill of the culture, sub-MIC concentration of BDQ (**Figure 4A**) or CCCP (**Figure 4B**) reduced the bactericidal effect of INH in a dose dependent manner up to 100-fold. The dose dependent increase of bacterial survival by BDQ or CCCP co-treatment was associated with a dose dependent suppression of the ATP burst (**Figures 4C,D**). To determine whether this finding extends to other cell wall synthesis inhibitors, we tested the effects of BDQ and CCCP on the bactericidal activity of EMB and found the same effects: co-treatment with EMB and inhibitors of oxidative phosphorylation at sub-MICs increased survival and reduced the ATP surge (**Figure 5**).

These results show that sub-inhibitory concentrations of oxidative phosphorylation inhibitors attenuate the bactericidal activity of cell wall synthesis inhibitors. Suppression of cell death is associated with suppression of drug induced ATP burst, suggesting that cell death is mediated in part by the ATP surge.

### Pharmacological Suppression of Drug-Induced ATP Burst Suppresses *iniBAC* Promoter Induction

Mycobacteria harbor the cell envelope stress response operon, *iniBAC*, which is transcriptionally induced by cell wall synthesis inhibitors, independent of their specific targets (Alland et al., 2000; Colangeli et al., 2005). Thus, anti-mycobacterials that cause an ATP burst also induce *iniBAC* expression. Interestingly, the IniR transcriptional activator controlling the expression of *iniBAC* harbors an AAA ATPase domain which may be involved in regulating its own activity (Boot et al., 2017). Given the apparent association between ATP burst and *iniBAC* induction, and the possibility that the *iniBAC* regulator may sense ATP via its ATPase domain, we hypothesized that increased ATP levels caused by cell wall synthesis inhibitors may represent a signal for *iniBAC* induction via IniR activation. If that is true, suppression of the ATP burst should suppress *iniBAC* induction.

To measure transcriptional activation of *iniBAC* we made use of a *M. bovis* BCG reporter strain harboring the promoter of *iniBAC* fused to the coding sequence of the red fluorescent protein, RFP (*M. bovis* BCG p*iniBAC*-RFP, (Yang T. et al., 2017)). Induction of p*iniBAC* increases the intrabacterial amount of RFP which can be measured as increased fluorescence of the cultures (Alland et al., 2000; Yang T. et al., 2017). To confirm that all drugs that cause an ATP burst indeed induce *iniBAC* expression we tested all 11 drugs used earlier for measurements of ATP levels, for their effect on p*iniBAC* activity. Exponentially growing *M. bovis* BCG p*iniBAC*-RFP cultures were treated for 24 h at MIC concentrations and RFU of the culture were measured as readout of p*iniBAC* promoter activity. As expected, all cell wall synthesis inhibitors caused increased fluorescence of cultures by 6 to 10-fold indicating induction of p*iniBAC* whereas all non-cell wall synthesis inhibitors did not affect p*iniBAC* activity (**Figure 1**). Thus, there was a strict correlation between drugs that trigger an increase of ATP concentration and p*iniBAC* promoter activity (**Figure 1**). To determine whether increase in p*iniBAC* activity depends on the increase of ATP levels, we co-treated *M. bovis* BCG p*iniBAC*-RFP cultures with INH and increasing sub-MIC concentration of BDQ or CCCP for 24 h and measured fluorescence of the cultures. Sub-inhibitory concentrations of BDQ or CCCP that abrogated the ATP surge also abrogated p*iniBAC* induction (**Figure 2**). The same results were obtained when INH was substituted with a different cell wall synthesis inhibitor, EMB (**Figure 3**).

Together, these results show that activity of the *iniBAC* cell envelope stress promoter follows the drug treatment dependent ATP concentration patterns: Drug induced ATP surge was associated with increased *iniBAC* promoter activity and pharmacological suppression of ATP increase suppressed *iniBAC* promoter activity. This suggests a link between ATP level and *iniBAC* expression. These results are consistent with a model in which ATP represents a signaling molecule for *iniBAC* induction. Whether the *iniBAC* regulator IniR is indeed the mechanistic link and senses the ATP burst via its AAA ATPase domain remains to be determined.

## Discussion

Antibacterials inhibit usually well-defined primary targets preventing the synthesis of some cell components. Studies over the past decade have shown that inhibition of an individual enzyme often appears to be insufficient in itself to cause rapid bacterial cell death. Rather, death is achieved by complex intracellular “corruption” events, which follow on from the initial target modulation by the drug, resulting, for instance, in accumulation of broadly toxic intermediates or suicidal derailing of signaling systems that ultimately cause collapse of central homeostatic systems within the bacteria (Dick and Young, 2011; Cho et al., 2014; Yang J.H. et al., 2017).

A key metabolite in bacterial physiology is ATP. Like all cells, bacteria need to maintain their intracellular ATP concentration within a certain range to maintain viability. In mycobacteria, reduction of intrabacterial ATP below a lower threshold is exploited by the anti-tuberculosis drug BDQ. BDQ is an inhibitor of F-ATP synthase and thus affects ATP levels directly by blocking regeneration of ATP via oxidative phosphorylation (Andries et al., 2005; Rao et al., 2008; Gengenbacher et al., 2010). Interestingly, Tomioka and colleagues showed that ATP has antimycobacterial activity when supplied exogenously. This suggests that excessive ATP can exert toxic effects. The mechanism underlying “high” ATP toxicity in mycobacteria is likely multifactorial. ATP is a cofactor for, and regulator of numerous cellular processes and metabolic pathways. Crossing an upper threshold could therefore cause dysregulation and malfunctioning of many pathways and networks. Furthermore, it was shown recently that high ATP levels, due to the metal chelating property of the nucleotide, can affect iron homeostasis and that this contributes to toxic effects of the nucleotide (Tatano et al., 2015).

Here, we asked whether increase in ATP to toxic levels may be part of the intracellular follow-on events triggered by some anti-mycobacterials. Surprisingly, we found that *all* cell wall synthesis inhibitors, but not other antibiotics tested, triggered a 4 to 5-fold increase in intrabacterial ATP concentration. This drug induced ATP burst could be suppressed by inhibitors of oxidative phosphorylation, suggesting that cell wall synthesis inhibitors trigger a common cell envelope stress response involving increased ATP production by oxidative phosphorylation. Importantly, pharmacological suppression of the ATP surge attenuated the kill effect of cell wall antibiotics, indicating that ATP increase contributes to the lethal effect of the drugs. Thus, “death by (excess) ATP” appears to be a strategy utilized by inhibitors of cell wall synthesis to kill mycobacteria.

Our finding is consistent with recent observations made in single cells analyses of *Mycobacterium smegmatis* (Maglica et al., 2015). In this study, INH treatment caused an increase in intracellular ATP in this fast-growing saprophytic *Mycobacterium*, suggesting that the ATP burst triggered by cell wall synthesis inhibition may be conserved across different mycobacterial species.

Interestingly, pharmacological suppression of drug-induced ATP surge also prevented induction of the cell envelope stress response operon *iniBAC* (Alland et al., 1998). The functions of the proteins of this gene cluster are unknown (Boot et al., 2017). The fact that these genes are strongly and specifically induced by cell wall synthesis inhibitors suggests that they have protective and/or repair functions involved in maintaining cell viability and envelope integrity (Boot et al., 2017). Recently, the transcription factor IniR was identified as an extremely specific inducer of *iniBAC* (Boot et al., 2017). This regulator senses the cell wall component trehalose, which is released upon cell envelope damaging events. Interestingly, the signal transducing IniR transcription factor also contains an AAA ATPase domain. It is tempting to speculate that this ATPase domain may play the role of ATP sensor in the regulation of IniR activity and thus explain the apparent dependence of *iniBAC* promoter induction on the intrabacterial ATP level.

## Conclusion

In conclusion, we show that “death by ATP” appears to be an extended mechanism of action employed by inhibitors of mycobacterial cell wall synthesis to exert their bactericidal activity. Interestingly, induction of the cell envelope stress operon *iniBAC* appears to require drug induced increase of ATP. Hence, this work reveals links between four processes, namely inhibition of cell wall synthesis, oxidative phosphorylation, cell death and *iniBAC* induction. The identification of the molecular mechanisms linking these processes, and how ATP burst contributes to cell death may reveal novel drug induced death pathways and targets that can be exploited for the discovery of bactericidal antibiotics. This work may also have clinical implications. Our *in vitro* data suggest antagonism between Bedaquiline and cell wall synthesis inhibitors. Hence, combining these two drug classes should be carefully considered. Co-treatment of patients with Bedaquiline and cell wall synthesis inhibitors may be counterproductive.

## Author Contributions

AS carried out the experiments. AS and TD designed the work and wrote the manuscript.

## Conflict of Interest Statement

The authors declare that the research was conducted in the absence of any commercial or financial relationships that could be construed as a potential conflict of interest.

## Abbreviations

INH: isoniazid
EMB: ethambutol
BDQ: bedaquiline
CCCP: carbonyl cyanide chlorophenylhydrazone, iniBAC, isoniazid inducible operon
